# RUNX1 突变急性髓系白血病患者临床特征分析

**DOI:** 10.3760/cma.j.cn121090-20250427-00202

**Published:** 2026-01

**Authors:** 雨轩 董, 文洁 戴, 婉秋 张, 蓓蓓 谢, 青 张, 毅 董, 慧 秦, 志敏 翟, 千山 陶, 莉莉 陶

**Affiliations:** 安徽医科大学第二附属医院血液科、血液病诊疗中心，安徽医科大学血液病研究中心，合肥 230601 Department of Hematology and the Diagnosis and Treatment Center for Hematologic Diseases of the Second Affiliated Hospital of Anhui Medical University, Anhui Medical University Hematology Research Center, Hefei 230601, China

## Abstract

回顾性分析2018年2月至2023年5月就诊于安徽医科大学第二附属医院的323例初诊急性髓系白血病（AML）患者的RUNX1突变情况和临床特征。AML患者中RUNX1的突变发生率为11.5％中位突变频率为40.4％；RUNX1突变常与ASXL1等突变共存，与NPM1突变共斥；合并RUNX1突变的AML患者中位年龄大（65岁对55岁，*P*<0.01），完全缓解率低（39.4％对79.8％，*P*<0.01），复发率（75.0％对48.8％，*P*<0.05）及死亡率（91.4％对59.5％，*P*<0.01）高，中位总生存期（5.9个月对20.1个月，*P*<0.01）及无复发生存（RFS）期（9.5个月对46.5个月，*P*<0.01）短；RUNX1的高突变频率与疾病的高死亡率（100.0％对76.5％，*P*<0.05）及更短的RFS期（7.2个月对12.2个月，*P*<0.05）相关；不同的诱导治疗方案（化疗/低强度治疗）对RUNX1突变患者的疗效和预后均没有显著影响（*P*>0.05）；RUNX1合并DNMT3A或ASXL1突变的患者预后更差，合并FLT3-ITD突变治疗反应差。

## Abstract (en)

We retrospectively analyzed the RUNX1 mutation status and clinical characteristics of 323 newly diagnosed acute myeloid leukemia（AML）patients treated in the Second Affiliated Hospital of Anhui Medical University from February 2018 to May 2023. The mutation rate of RUNX1 in AML patients was 11.5％ with a median mutation frequency of 40.4％. RUNX1 mutations often coexisted with other mutations, such as ASXL1, but were mutually exclusive with NPM1 mutations. AML patients with RUNX1 mutations were older（median age: 65 *vs* 55 years, *P*<0.01）, had a lower complete remission rate（39.4％ *vs* 79.8％ *P*<0.01）, higher relapse（75.0％ *vs* 48.8％, *P*<0.05）and mortality（91.4％ *vs* 59.5％, *P*<0.01）rates, shorter median overall survival（OS）（5.9 *vs* 20.1 months, *P*<0.01）and relapse-free survival（RFS）（9.5 *vs* 46.5 months, *P*<0.01）. A high RUNX1 mutation frequency was associated with increased mortality（100.0％ *vs* 76.5％, *P*<0.05）and shorter RFS（7.2 *vs* 12.2 months, *P*<0.05）. Different induction therapies（chemotherapy/low-intensity treatment）showed no significant impact on the efficacy or prognosis of patients with RUNX1 mutations（*P*>0.05）. Patients with concurrent RUNX1 and DNMT3A or ASXL1 mutations portend a poorer prognosis, and the cooccurrence of FLT3-ITD mutation further leads to inferior therapeutic outcomes.

Runt相关转录因子1（RUNX1）由位于人类21号染色体上的RUNX1基因编码，于1991年首次被发现，其作为重要的转录调节因子参与正常造血和恶性血液病的发生[1]–[2]。RUNX1突变发生在高达10％的骨髓增生异常综合征（MDS）和新发或继发性急性髓系白血病（AML）患者中[3]–[4]；并且可以促进MDS和骨髓增殖性肿瘤（MPN）的白血病转化[4]–[5]，在AML的发生中发挥重要调控作用。

近年来随着二代测序技术在血液肿瘤诊断、预后评估等方面的不断应用，已有多项研究证实RUNX1突变与AML的不良预后密切相关[3],[6]–[7]。本研究旨在通过纳入大样本AML患者的临床资料，分析AML患者中RUNX1的突变特征，探究RUNX1突变对疾病治疗效果、生存等方面的影响。

## 病例与方法

1. 病例：本研究回顾性分析了2018年2月至2023年5月期间就诊于安徽医科大学第二附属医院的323例初诊非M_3_型AML患者，所有患者均符合2016年WHO制定的AML诊断标准，并完成了详细的骨髓MICM评估检测及二代测序检查（其中二代测序检测经患者知情同意后由第三方医学检验公司完成）。

2. 治疗方案：AML患者的治疗均严格依据《成人急性髓系白血病（非急性早幼粒细胞白血病）中国诊疗指南（2017、2021、2023年版）》进行，对于适合强化疗的AML患者采用蒽环类药物联合阿糖胞苷方案进行诱导治疗；不适合强化疗的患者采用去甲基化药物联合/不联合BCL-2抑制剂、预激方案等低强度方案诱导治疗；完全缓解（CR）后根据患者的疾病危险度分层分别选择不同的缓解后治疗方案；复发难治性AML患者的后续治疗依据《中国复发难治性急性髓系白血病诊疗指南（2017、2021、2023年版）》推荐方案进行，所有AML患者接受的诱导治疗具体方案见表1。

**表1:** 纳入研究的323例急性髓系白血病患者的具体治疗方案（例）

治疗方案	RUNX1突变组（37例）	RUNX1未突变组（286例）
标准化疗		
IA	16	179
DA	0	12
HA	3	13
化疗+去甲基化药物^a^		
IA+地西他滨	2	21
IA+阿扎胞苷	0	8
IA+维奈克拉^b^	2	3
去甲基化药物单药^c^		
阿扎胞苷	0	9
地西他滨	0	6
去甲基化药物+维奈克拉^d^		
阿扎胞苷+维奈克拉	5	15
地西他滨+维奈克拉	3	14
预激方案（CAG）	5	4
未治疗	1	2

**注** IA：去甲氧柔红霉素+阿糖胞苷；DA：柔红霉素+阿糖胞苷；HA：高三尖杉酯碱+阿糖胞苷；CAG：阿糖胞苷+阿克拉霉素+G-CSF；^a^标准IA方案基础上联合地西他滨15 mg/m^2^×5 d或阿扎胞苷75 mg/m^2^×7 d；^b^标准IA方案基础上联合维奈克拉（100 mg第1天，200 mg第2天，400 mg第3～28天）；^c^阿扎胞苷75 mg/m^2^×7 d或地西他滨15 mg/m^2^×5 d；^d^阿扎胞苷75 mg/m^2^×7 d或地西他滨15 mg/m^2^×5 d联合维奈克拉（100 mg第1天，200 mg第2天，400 mg第3～28天）

3. 疗效及预后评估标准：诱导治疗结束后21～28 d左右复查骨髓，CR定义为骨髓原始细胞<5％无外周血原始细胞，髓外病灶消失，中性粒细胞绝对数≥1.0×10^9^/L，PLT≥100×10^9^/L。总生存（OS）时间定义为从初诊到死亡或末次随访的时间；无复发生存（RFS）时间定义为从CR后到疾病首次复发的时间。主要观察指标为疾病的OS，首疗程诱导治疗的CR率；次要指标为疾病整体死亡率、复发率、RFS。

4. 临床资料收集：收集所有纳入患者的基本信息（年龄、性别）及相关临床资料（包括FAB分型、初诊时原始细胞比例、血常规水平、骨髓MICM资料及二代测序结果、接受的诱导治疗方案、首疗程诱导治疗后疗效，以及OS、RFS等）。

5. 随访：所有的患者后续均以定期门诊就诊、住院检查或电话随访等方式接受规律的随访，所有AML患者的中位随访时间为14.7（0.1～67.8）个月，其中RUNX1突变组为5.0（0.1～65.1）个月，RUNX1未突变组为16.1（0.7～67.8）个月。

6. 统计学处理：所有数据均采用SPSS 26.0和GraphPadPrism 9.0.2进行统计学分析。定量资料符合正态分布使用均数±标准差进行表示，组间比较采用*t*检验；不符合正态分布采用中位数（范围）进行表示，组间比较采用非参检验。定性资料以频数或频率进行描述表示，采用卡方检验或Fisher精确检验进行组间比较。生存曲线采用Kaplan-Meier法绘制，组间RFS和OS的比较采用对数秩检验；不同基因突变之间共存或互斥的线性关联关系使用Pearson相关系数（*r*）表示，*P*<0.05为差异有统计学意义。

## 结果

1. RUNX1突变概况：在纳入的323例AML患者中共有37例患者检出RUNX1突变，其突变发生率为11.5％，突变频率波动于1.1％～89.2％，中位突变频率为40.4％；相较于RUNX1未突变组，合并RUNX1突变的AML患者中位年龄偏大（65岁对55岁，*P*<0.01）；RUNX1突变与患者性别、疾病的FAB分型、初诊原始细胞比例、血细胞水平等没有显著的相关性。两组患者的临床特征详见表2。

**表2:** 纳入的323例急性髓系白血病患者的临床特征

特征	RUNX1突变（37例）	RUNX1未突变（286例）	*P*值
年龄［岁，*M*（范围）］	65.0（24.0~84.0）	55.0（15.0~82.0）	<0.01
性别［例（％）］			0.21
男	22（59.5）	138（48.3）	
女	15（40.5）	148（51.7）	
FAB分型［例（％）］			0.86
M_0_	1（2.7）	0（0）	
M_1_	1（2.7）	18（6.3）	
M_2_	18（48.6）	107（37.4）	
M_4_	1（2.7）	39（13.6）	
M_5_	7（18.9）	61（21.3）	
M_6_	0（0）	1（0.3）	
Mu	9（24.3）	60（21.0）	
原始细胞［％，*M*（范围）］	49.3（20.0~92.5）	60.0（3.5~97.0）	0.09
WBC［×10^9^/L，*M*（范围）］	5.60（0.57~332.50）	14.63（0.40~440.00）	0.21
HGB［g/L，*M*（范围）］	75（41~136）	75（32~138）	0.94
PLT［×10^9^/L，*M*（范围）］	36（3~224）	40（3~485）	0.87
接受移植［例（％）］			0.32
是	5（13.5）	61（21.3）	
否	30（81.1）	223（78.0）	
失访/死亡（例）	2（5.4）	2（0.7）	

所有RUNX1突变的AML患者均被检出至少合并1种其他基因突变；其中4例（10.8％）患者仅携带1个共突变基因，6例（16.2％）携带2个共突变基因，27例（73.0％）携带3个及以上共突变基因，所有RUNX1突变的AML患者合并突变的情况见图1；其中合并的突变基因按照发生频率排序从高到低分别为：ASXL1（35.1％），DNMT3A（32.4％），IDH2、TET2（29.7％），SRSF2（21.6％），FLT3-TKD（18.9％），BCORL1、FLT3-ITD（16.2％），BCOR、CSF3R、IDH1、NRAS、PTPN11、U2AF1、ZRSR2（10.8％），CBL、EZH2、TP53（8.1％），CEBPA、JAK2、PHF6、SF3B1、STAG2、WT1（5.4％），ASXL2、CALR、CALRL、GATA2、IKZF3、KIT、NF1、NOTCH1、NOTCH3（2.7％）；此外我们发现RUNX1基因在AML患者中往往与ASLX1、CALR、BCORL1、FLT3-TKD、SRSF2、ZRSR2、EZH2、STAG2、U2AF1、CSF3R等基因突变共存，与NPM1突变互斥（*P*<0.05）。

**图1:**
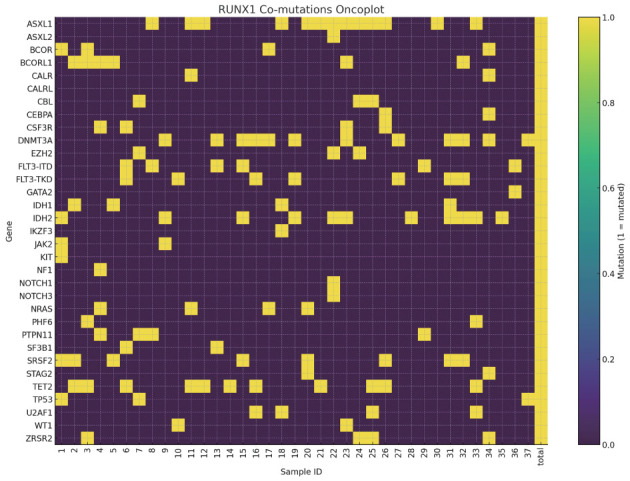
37例急性髓系白血病（AML）患者RUNX1共突变情况 **注** 采用Oncoplot图详细展示了所有RUNX1突变AML患者共突变情况；图中每一纵列代表每一例RUNX1突变的AML患者，每一横列表示突变基因，黄色表示存在该基因突变

2. RUNX1突变对治疗效果和疾病预后影响：在治疗效果上，合并RUNX1突变的AML患者相较RUNX1未突变组首疗程诱导治疗后CR率更低（39.4％对79.8％，*P*<0.01），死亡率（91.4％对59.5％，*P*<0.01）、复发率（75.0％对48.8％，*P*＝0.04）更高；在长期预后方面，合并RUNX1突变的AML患者中位OS期仅为5.93个月，中位RFS期仅为9.55个月；相较RUNX1未突变的患者中位OS期（20.10个月）及RFS期（46.53个月）均明显缩短，差异均具有统计学意义（图2A、2B）。

**图2:**
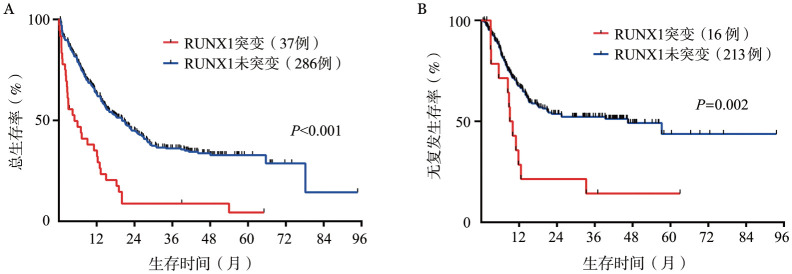
RUNX1突变对急性髓系白血病患者总生存（OS）和无复发生存（RFS）的影响

3. RUNX1突变AML患者对不同治疗方案的反应：在合并RUNX1突变的AML患者中，接受标准化疗或低强度治疗对疾病的CR率（42.9％对33.3％，*P*＝0.59）、死亡率（91.0％对92.3％，*P*＝0.89）、复发率（75.0％对75.0％，*P*＝1.00）均没有明显的影响；此外在中位OS期（5.00个月对5.93个月，*P*＝0.98）和中位RFS期（8.64个月对12.20个月，*P*＝0.24）上差异也无统计学意义。37例RUNX1突变患者中共有10例（低强度治疗组8例，标准化疗组2例）在首疗效诱导治疗时使用了维奈克拉；统计分析表明，诱导治疗方案中是否含有维奈克拉患者组的CR率（50.0％对36.0％，*P*＝0.48）、死亡率（77.8％对96.2％，*P*＝0.09）、复发率（50.0％对83.3％，*P*＝0.18）、中位OS期（4.40个月对6.47个月，*P*＝0.97）及RFS期（9.10个月对10.00个月，*P*＝0.62）差异均无统计学意义。

4. RUNX1突变频率对AML的影响：依据纳入患者RUNX1的中位突变频率（40.4％）将所有合并RUNXI突变的AML患者分为RUNX1高频突变组（19例）和RUNX1低频突变组（18例）。统计分析发现，两组间在原始细胞的比例上差异无统计学意义［（54.0±24.2）％对（46.4±20.8）％，*P*＝0.32）］；在治疗效果方面，RUNX1高频突变组的死亡率高于RUNX1低频突变组（100.0％对76.5％，*P*＝0.02），而在首疗程CR率（26.3％对47.1％，*P*＝0.20）、复发率（85.7％对70.0％，*P*＝0.45）上两组间差异无统计学意义；在预后方面，RUNX1高频突变组中位RFS期显著短于RUNX1低频突变组（7.18个月对12.20个月，*P*＝0.03）（图3A），但在中位OS期上两组间差异无统计学意义（5.93个月对11.17个月，*P*＝0.30）（图3B）。

**图3:**
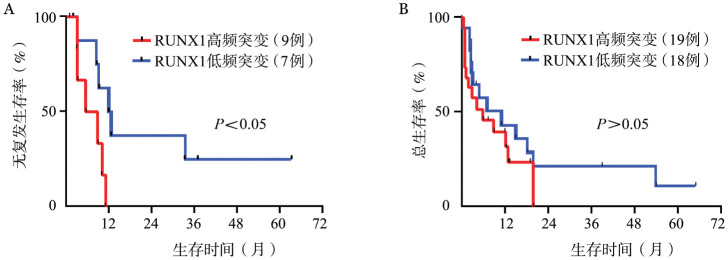
RUNX1突变频率对急性髓系白血病患者无复发生存（A）和总生存（B）的影响

5. RUNXI合并其他基因突变对疗效和预后的影响：RUNX1合并DNMT3A突变AML患者（12例）的中位OS期仅为2.90个月，中位RFS期仅为3.13个月，相较于未合并DNMT3A突变患者（25例）的中位OS期11.17个月及RFS期11.00个月均显著缩短（*P*＝0.02，*P*＝0.02）（图4A、4B），并且疾病的死亡率更高（100.0％对87.0％，*P*＝0.01）；但是在疾病的首疗程CR率（36.4％对40.9％，*P*＝0.80）及复发率（75.0％对75.0％，*P*＝1.00）差异无统计学意义。在RUNX1合并ASXL1突变AML患者中（13例），我们同样发现疾病中位OS期的显著缩短（3.07个月对11.17个月，*P*＝0.03）（图C）；此外我们发现，RUNX1合并FLT3-ITD突变的患者（6例）首疗程CR率更低（0对48.2％，*P*＝0.03），但是否合并FLT3-ITD突变对疾病的死亡率（100.0％对89.7％，*P*＝0.41）、复发率（100.0％对73.3％，*P*＝0.55）、中位OS期（4.13个月对7.00个月，P＝0.58）及RFS期（10.00个月对9.10个月，*P*＝0.81）均没有明显的影响。

**图4:**
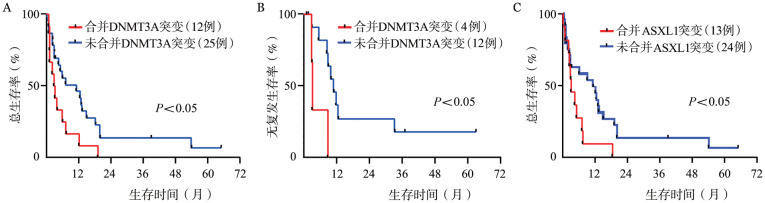
RUNX1合并DNMT3A（A、B）、ASXL1（C）突变对急性髓系白血病患者预后的影响

## 讨论

RUNX1基因作为重要的转录调节因子在血细胞分化的过程中发挥重要的调节作用。RUNX1突变可导致造血祖细胞（HPC）和髓系祖细胞扩增异常，表现为细胞分化受损、对遗传毒性抵抗力减低，蛋白质翻译受抑，核糖体生物合成减少等，从而参与AML及其他多种肿瘤的发生[2],[8]。多种血肿瘤患者均存在RUNX1突变，在MDS患者中其突变发生率约为10％在慢性粒-单核细胞白血病（CMML）患者中约为37％[9]，而在AML患者中约为10％[4]，这与我们的研究结果相符。RUNX1在AML患者中大多为功能丧失性突变，从而影响髓系祖细胞的分化，介导细胞对化疗的耐药，与AML的不良预后密切相关；近期一项大样本研究报道RUNX1突变的AML患者首次诱导治疗CR率仅为19.6％总体反应率仅为73.5％5年OS仅为35.4％相较于RUNX1未突变组（CR率、总反应率、5年OS率分别为29.7％、84.3％、42.3％）均显著降低[10]，也有部分研究显示RUNX1突变对AML的预后可能没有显著影响[11]–[12]。我们的研究表明RUNX1突变可导致AML患者诱导治疗CR率降低，中位OS期和RFS期显著缩短，该结果与国内外大多数研究基本一致，充分证实RUNX1突变与疾病的不良预后密切相关；此外，我们还发现RUNX1的高突变频率可导致疾病的死亡率更高，RFS期更短。尽管目前国内外多项指南已将RUNX1突变纳入AML的不良分子生物学预后标志，但对RUNX1的突变频率未做特殊的强调和要求，对于RUNX1突变频率是否影响疾病的疗效及预后也暂缺乏明确的报道。

既往研究表明RUNX1突变在AML患者中往往与FLT3、DNMT3A、ASXL1、NRAS、KIT和IDH1/2等突变同时发生，与NPM1或CEBPA突变相互排斥[3]–[4],[13]；此外，RUNX1合并ASXL1、DNMT3A、EZH2等共突变可能导致疾病预后更差[12],[14]。我们的研究同样发现RUNX1突变常与ASXL1等多种基因突变共存，与NPM1突变互斥；此外我们发现RUNX1合并DNMT3A突变可导致疾病中位OS期及RFS期更短，死亡率更高；合并ASXL1突变同样会导致疾病的OS期更短；合并FLT3-ITD突变则导致疾病的CR率更低；DNMT3A突变的AML患者对去甲基化药物更加敏感，但疾病的OS和预后往往更差[15]；ASXL1突变同样与AML的不良预后密切相关[6]；因此我们推测除了RUNX1突变本身对疾病的影响，其伴随的其他多种不良分子生物学标志可能共同参与疾病的进程，导致疾病的不良预后。

RUNX1突变的AML患者对治疗反应较差，缺乏特定的靶向治疗靶点以及对化疗的耐药性可能是其疗效不佳的重要原因[1],[6]。我们的研究发现，无论是采用蒽环类药物联合阿糖胞苷的强化疗方案，还是采用去甲基化药物联合/不联合BCL-2抑制剂、预激方案等低强度治疗方案，均不能改善疾病的疗效及预后。近期有研究表明伴RUNX1突变的AML细胞对高三尖杉酯碱（HHT）和BCL-2抑制剂可能更加敏感。HHT通过降低抗凋亡蛋白c-Myc、MCL-1和Bcl-xL的水平导致肿瘤细胞对BCL-2的依赖性增加，在此基础上连用BCL-2抑制剂维奈克拉可以显著增加抗肿瘤效应[16]；此外，近期有研究表明去甲基化药物和维奈克拉的联合用药可改善RUNX1突变患者的预后[3],[17]；然而我们的研究发现在诱导治疗方案中加入维奈克拉并不能提升疾病的缓解率并改善预后；这可能与我们研究中接受维奈克拉治疗的患者相对较少、未选用合适的联合用药方案相关。总体来说，目前对于RUNX1突变的AML患者缺乏有效的治疗方案，需要在未来进一步探索。

近期有研究表明RUNX1突变对疾病的不良影响可以通过CR1后的移植来克服[18]。我们的研究中共有5例RUNX1突变AML患者在CR1后接受了allo-HSCT治疗，其中4例患者最终复发并死亡，中位OS期仅为13.30个月，相较于未接受移植的RUNX1突变AML患者在疾病的复发率、死亡率以及OS均上没有明显差异，这可能与我们研究中接受allo-HSCT的患者较少相关。

总之，本研究证实了RUNX1突变与AML的不良预后相关，该类患者缺乏有效的治疗方案，死亡率及复发率高，OS期短，在未来需要更加有效的治疗手段改善疾病预后。
